# Editorial: Virtual reality and empathy

**DOI:** 10.3389/fpsyg.2022.1089006

**Published:** 2022-11-21

**Authors:** Sofia Seinfeld, Béatrice S. Hasler, Domna Banakou, Jonathan Levy

**Affiliations:** ^1^Image Processing and Multimedia Technology Center, Universitat Politècnica de Catalunya-Barcelona Tech, Terrassa, Spain; ^2^Sammy Ofer School of Communications, Reichman University, Herzliya, Israel; ^3^Interactive Media Program, Arts and Humanities Division, New York University Abu Dhabi, Abu Dhabi, United Arab Emirates; ^4^Department of Neuroscience and Biomedical Engineering, Aalto University, Espoo, Finland; ^5^Baruch Ivcher School of Psychology, Reichman University, Herzliya, Israel

**Keywords:** virtual reality, empathy, perspective-taking, immersion, embodiment

One of the greatest potentials of immersive VR experiences, envisioned by broad sectors of the scientific community as well as the industry, is its use to increase empathy toward different types of populations and situations (Louie et al., 2018; van Loon et al., 2018; Modena and Pinotti, 2020). Some common ways in which VR has been used to enhance empathy are: (1) through the simulation of intergroup encounters, (2) by providing a bystander perspective of discriminative behaviors, or (3) by enabling participants to experience discriminative behaviors from the (embodied) perspective of an outgroup member or victim. There is evidence that some of these VR experiences might lead to positive outcomes, such as reducing implicit racial bias (Peck et al., 2013; Banakou et al., 2016, 2020; Hasler et al., 2017; Bedder et al., 2019), enhancing emotion recognition in relation to victims of violent behaviors (Hamilton-Giachritsis et al., 2018; Seinfeld et al., 2018, 2022; Ventura et al., 2021), or increasing empathy toward people with disabilities (Dyer et al., 2018; Brydon et al., 2021; Chowdhury and Quarles, 2021). Nonetheless, there are also studies reporting mixed findings, with VR not always leading to enhancements in empathy (Hasler et al., 2021), and even resulting in negative effects in certain cases (Groom et al., 2009; Sundar et al., 2017; Weinel et al., 2018; Banakou et al., 2020; Ventura et al., 2020; Martingano et al., 2021; Ho and Ng, 2022). For example, Banakou et al. (2020) reported that negative affective VR simulations might result in an increase of implicit racial bias, supporting earlier findings by Groom et al. (2009), and a recent meta-analysis indicates that VR increases emotional components of empathy, rather than the cognitive ones (Martingano et al., 2021). Based on a review of studies, Bloom (2017) concludes that empathy does not lead to behavioral changes and argues instead for rational compassion.

Such divergent results could be partially explained by the different types of methodologies employed, design factors, technologies used, and the virtual content itself implemented to address empathy, and variations in factors such as instructions (i.e., empathetic or in-his-shoes perspective-shifting), realism (i.e., computer-graphics vs. 360-degree videos), immersiveness (i.e., higher or lower), degree of interactivity (i.e., being able to modify the content or not), and visual perspective (i.e., 1PP with or without a virtual body, or bystander perspective). Recently, authors with diverse theoretical backgrounds have also started introducing and discussing the ethical implications of using VR as an empathy-enhancing tool (Rueda and Lara, 2020; Slater et al., 2020; Slater and Banakou, 2021; see Ramirez et al., 2021). Nakamura (2020) refers to the empathy arising through VR experiences as “toxic empathy” that only allows privileged people to claim that they have experienced what life is like for the disadvantaged population, but does not lead to changes in people's behavior.

The mixed results and perspectives on the use of VR as an empathy machine show that the impact of the technology, on such a multidimensional and complex concept as empathy, is far from being straightforward. However, this evidence also emphasizes the need for further research and evidence-based discussions in relation to the consequences, applications, and VR design factors that can influence the use of this technology as a potential “empathy-machine”. The present Research Topic, *Virtual reality and empathy*, aims to contribute to this debate by presenting experimental studies, reviews, and critical opinions in relation to this issue.

As part of this article Research Topic, Sora-Domenjó reviews several experimental studies, as well as immersive media experiences, to discuss some of the benefits, risks, and ethical concerns raised by the use of VR in addressing social issues through storytelling and embodiment. This interdisciplinary work concludes that there is no sufficient evidence to claim that VR exposure *per se* is able to increase empathy and enhance prosocial behaviors toward stigmatized groups. Despite the claims of several film directors and journalists that immersive media can enhance empathy toward underprivileged members, the author demonstrates that there is a concerning lack of research supporting such statements, and appeals to the need for further rigorous scientific research to better understand the short and long-term consequences of immersive media and film VR experiences.

On a different view, in an opinion article, Lara and Rueda challenge Ramirez et al. (2021) ethical rejection of empathy enhancement through VR arguing that in this article there is a misunderstanding of the conception of using VR to “imagine-other” vs. “imagine-self” (Goldie, 2011). Ramirez et al. (2021) argue that it is impossible and unethical to use VR to simulate being another person due to real-life interpersonal disparities and the failure to access someone else's subjectivity (e.g., cognition, attitudes, personality, etc.), concluding that VR is only beneficial for sympathy-based interventions. However, Lara and Rueda argue that VR can be a very powerful tool to enhance empathy when it is not used to merely pretend to be someone else (i.e., assuming it is impossible to enter into someone else's identity and mind), but rather to seek to get a personal idea of the thoughts and feelings you would experience if you were in the other person's circumstances. They argue that this is effectively achieved through embodiment in VR and proper instructions before going into the VR experience, citing a body of research literature that supports their view.

With respect to the use of embodiment in VR as a potential resource to facilitate empathy toward stigmatized groups, as part of the present article Research Topic, Marques et al. describe a study where they explore how experiencing psychotic symptoms while performing a cognitive task in VR can impact empathy, knowledge, and attitudes toward people with schizophrenia. They report a study conducted with higher education health students and compare their results to only watching these symptoms on a 2D screen. Interestingly, the authors found that both VR and 2D videos seem to be useful tools to enhance empathy toward people with schizophrenia, with VR eliciting more effective attitudes and knowledge-based changes compared to the 2D screen condition.

Similarly, this article Research Topic also includes an experimental study realized by Tassinari et al., where the authors leverage a widely used commercial social VR platform, AltspaceVR, to better understand how positive intergroup contact with an avatar of a stigmatized outgroup (i.e., different race) impacts situational empathy. Despite participants experiencing the illusion of being embodied in their avatar while interacting with other avatars, the authors did not find evidence that positive intergroup contact (i.e., equal group status in a collaborative task) had an effect on empathy measured through self-report questionnaires. However, there were some indications that the experienced level of co-presence in the intergroup contact condition modulated the increase of post-intervention empathic feelings, an aspect that deserves further research in future studies.

To conclude, in accord with past studies, the experimental evidence, reviews, and discussions included in this article Research Topic, confirm that the use of VR to enhance empathy might not be as straightforward as initially assumed, and demands further investigation through rigorous scientific methods in order to better understand under what conditions VR technologies can be beneficial, but also detrimental. It may also be that mediating parameters such as personality traits, culture or political ideology may moderate effects on empathy. In order to move away from the concept of VR as an “empathy machine”, which has been scrutinized by many due to its ineffectiveness to lead to changed behavior, authors are introducing new paradigms toward enhancing prosocial behavior rather than simply changing empathy (Slater and Banakou, 2021).

## Author contributions

SS and BH drafted the manuscript. DB and JL critically revised the manuscript for important intellectual content. All authors conceived, designed the manuscript, and approved the final version of the manuscript.

## Funding

BH was funded by Israel Science Foundation (Grant No. 3105/21) and the Academy of Finland Research Fellow funding supported JL.

## Conflict of interest

The authors declare that the research was conducted in the absence of any commercial or financial relationships that could be construed as a potential conflict of interest.

## Publisher's note

All claims expressed in this article are solely those of the authors and do not necessarily represent those of their affiliated organizations, or those of the publisher, the editors and the reviewers. Any product that may be evaluated in this article, or claim that may be made by its manufacturer, is not guaranteed or endorsed by the publisher.
